# Prognostic Role of Kidney Disease in Newly Diagnosed Acute Myeloid Leukemia Under Venetoclax-Based Low-Intensity Therapy

**DOI:** 10.3390/cancers17182993

**Published:** 2025-09-13

**Authors:** Katja Krüger, Razif Gabdoulline, Martin Wichmann, Bernhard M. W. Schmidt, Katharina Götze, Krischan Braitsch, Laura Schmalbrock, Lars Bullinger, Franziska Westendorf, Walter Fiedler, Anke K. Bergmann, Jürgen Krauter, Stephan Kaun, Andreas Voß, Elisabeth Koller, Ulrich Germing, Kai Wille, Martin Grießhammer, Jan Braess, Daniel Föhring, Peter Reimer, Ulrich Kaiser, Heinz Kirchen, Frank Hartmann, Jan M. Middeke, Christoph Röllig, Hartmut Döhner, Konstanze Döhner, Gernot Beutel, Felicitas R. Thol, Florian H. Heidel, Michael Heuser, Rabia Shahswar

**Affiliations:** 1 Department of Hematology, Hemostasis, Oncology, and Stem Cell Transplantation, Hannover Medical School, 30625 Hannover, Germany; 2 Department of Nephrology and Hypertension, Hannover Medical School, 30625 Hannover, Germany; 3 Department of Medicine III, School of Medicine and Health, Technical University of Munich (TUM), 81675 Munich, Germany; 4 Department of Hematology, Oncology, and Cancer Immunology, Campus Benjamin Franklin, Charité-Universitätsmedizin Berlin, 12203 Berlin, Germany; 5 Department of Oncology, Hematology and Bone Marrow Transplantation with Section Pneumology, University Medical Center Hamburg-Eppendorf, 20251 Hamburg, Germany; 6 Department of Human Genetics, Hannover Medical School, 30625 Hannover, Germany; 7 Clinical Genetics and Genomic Medicine, University Hospital Würzburg, 97078 Würzburg, Germany; 8 Department of Internal Medicine, Municipal Hospital Braunschweig, 38126 Braunschweig, Germany; 9 Department of Internal Medicine, Municipal Hospital Bremen-Mitte, 28205 Bremen, Germany; 10 Department of Oncology and Hematology, University Clinic Oldenburg, 26133 Oldenburg, Germany; 11 3rd Medical Department, Hanusch Hospital, 1140 Vienna, Austria; 12 Department of Hematology, Oncology and Clinical Immunology, Heinrich Heine University Düsseldorf, 40225 Düsseldorf, Germany; 13 University Clinic for Haematology, Oncology, Haemostaseology and Palliative Care Johannes-Wesling-Klinikum Minden, Ruhr-University Bochum, 32429 Minden, Germany; 14 Department of Oncology and Hematology, Hospital Barmherzige Brueder Regensburg, 93049 Regensburg, Germany; 15 Evang. Krankenhaus Essen-Werden, 45239 Essen, Germany; 16 Medical Department II, St. Bernwards Hospital, 31134 Hildesheim, Germany; 17 Department of Internal Medicine I, Krankenhaus der Barmherzigen Brüder, 54292 Trier, Germany; 18 Department of Hematology and Oncology, Hospital Lippe, 32657 Lippe, Germany; 19 Department of Internal Medicine I, University Hospital Dresden, Technical University Dresden, 01307 Dresden, Germany; 20 Department of Internal Medicine III, University Hospital of Ulm, 89081 Ulm, Germany; 21 Department of Internal Medicine IV, University Hospital Halle (Saale), Martin-Luther-University Halle-Wittenberg, 06120 Halle, Germany

**Keywords:** acute myeloid leukemia, chronic kidney disease, acute kidney injury, venetoclax, treatment outcomes

## Abstract

Chronic kidney disease (CKD) before treatment and renal function decline during treatment are common in elderly patients receiving hypomethylating agents (HMAs) and venetoclax (VEN). This retrospective multicenter study of 130 newly diagnosed older acute myeloid leukemia (AML) patients evaluated the prognostic impact of renal function before and during the first treatment cycle. A total of 56 patients (43%) had CKD, and 49 (38%) developed acute kidney injury (AKI) during treatment. AKI occurrence was associated with laboratory tumor lysis syndrome (LTLS). CKD before treatment showed a trend towards a lower overall response rate (ORR, OR 0.5, *p* = 0.07) in multivariable analysis (MVA) and inferior relapse-free survival (RFS, HR 2.16, *p* = 0.06) in univariate analysis (UVA), but not MVA (RFS, HR 1.93, *p* = 0.15). CKD did not affect overall survival (OS) or event-free survival (EFS). AKI during HMA/VEN therapy was associated with significantly higher 30-day and 60-day mortality rates and emerged as an independent prognostic factor for inferior OS (HR 1.86, *p* = 0.01) and EFS (HR 1.81, *p* = 0.007). RFS did not differ significantly by AKI status. Sepsis was a more frequent cause of death in patients with vs. without AKI (33% vs. 5%). In conclusion, kidney function is a key prognostic factor in HMA/VEN-treated patients, warranting further study on treatment adjustments and supportive care.

## Simple Summary

For elderly patients diagnosed with acute myeloid leukemia, hypomethylating agents combined with venetoclax are often the treatment of choice. This less intensive treatment regimen compared to standard chemotherapeutic agents results in higher tolerability. However, the incidence of kidney impairment increases with age and is a common condition in this patient group. Its impact on outcomes remains unclear. In this retrospective study, we examined how pre-existing and newly developed kidney disease during treatment affect survival. Acute kidney injury during therapy was associated with a higher early mortality and shortened overall survival, suggesting that close monitoring and management of kidney function could help optimize patient outcomes. Chronic kidney disease prior to treatment had no significant effect on the patients’ survival or response to therapy, indicating that this treatment regimen can be applied in patients with pre-existing kidney disease.

## 1. Introduction

Based on favorable response and survival data reported in the Viale-A trial, treatment with HMA/VEN has been established as a standard of care in patients ineligible for intensive chemotherapy [1]. Median overall survival times ranging from 11.0 to 13.4 months and rates of complete remission (CR) or complete remission with incomplete blood count recovery (CRi) between 43% and 66.7% have been observed in the real-world treatment setting [2,3,4,5]. Recently, mutations in *TP53, FLT3*-ITD, *NRAS*, and *KRAS* were found to be associated with an inferior survival [6,7,8,9]. Further identified unfavorable risk factors include presence of an adverse karyotype (KT) and absence of an IDH2 mutation [10]. NPM1 mutations were described as being associated with superior survival and prolonged duration of remission [11]. However, little is known about non-genetic parameters and their impact on treatment outcomes. In a previous study, occurrence of treatment-emergent cardiac events resulted in a significantly shorter survival [12]. In contrast, renal insufficiency, a common comorbidity in elderly patients, has not been evaluated so far. Whether acute or chronic kidney injury may impact the management of HMA/VEN treatment of these patients remains unknown. 

VEN is primarily cleared by hepatic metabolism. Renal elimination plays a minor role, with <0.1% of administered VEN being excreted by this pathway [13]. Nonetheless, renal insufficiency might affect the pharmacokinetics (PKs) of VEN, as altered PKs can also be observed in drugs that are mainly degraded and eliminated by hepatic metabolism [14]. Few case reports regarding AML patients with CKD receiving VEN treatment are available. In one patient undergoing dialysis azacitidine (AZA)/VEN was described as an effective and feasible treatment option, though the dose of VEN was reduced [15]. Brackman et al. conducted population pharmacokinetic modeling, demonstrating similar VEN exposure regardless of renal impairment, suggesting that dose adjustments may not be necessary [16]. In addition, a study with six female patients with an estimated glomerular filtration rate (eGFR) <15 mL/min observed no effect of hemodialysis or renal impairment on PK of VEN [17]. Studies involving larger cohorts are needed to clarify the treatment of patients with renal insufficiency and its impact on outcomes. 

CKD affects 13.4% of the global population [18]. Common risk factors for developing CKD include a previous glomerulonephritis, hypertension and cardiovascular risk factors, such as obesity and diabetes [19,20,21,22]. AKI affects 21.6% of hospitalized patients. The risk of developing AKI increases in individuals with pre-existing CKD. Additional contributing risk factors include conditions such as sepsis, hypovolemia and hypotension or exposure to nephrotoxic contrast media or medications [23,24]. Furthermore, clonal hematopoiesis of indeterminate potential (CHIP) significantly raises the risk of developing AKI, particularly severe cases that require dialysis. This risk is most pronounced in individuals with mutations in genes such as *TET2, JAK2, ASXL1, PPM1D, TP53*, and *SRSF2*. Large CHIP clones, reflected by a high variant allele fraction, increase the likelihood for persistent CKD following AKI [25]. In cancer patients, tumor lysis syndrome (TLS) significantly increases the risk of renal insufficiency [26]. In a comparative analysis Otiker et al. analyzed the potential effect of HMA treatment on kidney function. Aza was more likely to induce AKI than decitabine, though overall, patients receiving HMA monotherapy are unlikely to experience renal impairment [27]. Importantly, HMAs have also been described as a feasible treatment option in patients with pre-existing CKD [28]. However, the risk of AKI is likely to be higher in combination regimens, given their potential for increased toxicity.

CKD results in increased serum creatinine levels, which proved to be a biomarker for the occurrence of TLS [29]. TLS, as a result of rapid degradation of malignant cells, can be differentiated into laboratory TLS (LTLS) with altered levels of uric acid, potassium, phosphorous and calcium, and clinical TLS (CTLS), with additional clinical symptoms such as oliguria, anorexia, vomiting, cramps, and seizures, besides others. TLS is commonly classified by the Cairo-Bishop definition [30]. Previous studies found LTLS rates ranging from 12% to 18% and CTLS rates between 2% and 5% in VEN-treated AML patients [31,32,33]. However, the number of reported patients experiencing acute kidney injury (AKI) related to TLS is limited, and no data are available on the role of isolated AKI during VEN treatment. This results in a lack of information regarding the clinical management of these patients.

This study aims to evaluate the feasibility of VEN-based non-intensive treatment regimens in patients with acute or chronic kidney disease and assess their prognostic impact for response and survival.

## 2. Materials and Methods

### 2.1. Patients

Patients were included in this analysis if they were ≥18 years of age and had a diagnosis of AML according to the 2022 International Consensus Classification of Myeloid Neoplasms and Acute Leukemias (ICC), excluding acute promyelocytic leukemia [34]. All patients received VEN-based non-intensive treatment regimens as first-line treatment and were reported to the venetoclax registry (venreg.org; ClinicalTrials.gov NCT03662724), a multicenter, retrospective and prospective, observational cohort study. VENreg was approved by the local Ethics Review Committee (ethical vote No.7972_BO_K_2018) [35,36]. This analysis involves patients from academic and outpatient centers in Germany and Austria, who received their first treatment cycle between January 2016 and April 2024. Written informed consent was obtained from all patients in accordance with the Declaration of Helsinki. The data cut-off for this analysis was 30 April 2024.

### 2.2. Treatment Administration

All patients received VEN-based non-intensive treatment regimens. VEN was administered once daily and was combined with azacitidine 75 mg/m^2^ days 1–7 or 1–5 and 8 + 9 subcutaneously (*n* = 111), decitabine 20 mg/m^2^ days 1–5 intravenously (*n* = 11) or low-dose cytarabine (LDAC) 20 mg twice daily days 1–10 subcutaneously (*n* = 6). VEN doses ranged from 50 mg to 600 mg, with dose reductions applied when CYP3A inhibitors were administered concomitantly. The duration of VEN treatment was <14 days (*n* = 17), 14 days (*n* = 43) or 28 days (*n* = 60). 

### 2.3. Cytogenetic and Molecular Analysis

Samples for genetic analysis were acquired from peripheral blood (if blasts were increased in blood) or bone marrow before treatment initiation with VEN. Cytogenetic and molecular analyses were performed as previously described [37,38,39]. 

### 2.4. Safety and Efficacy Assessment

The overall response rate (ORR) was defined as CR or CRi. CR and CRi were defined according to the 2022 European LeukemiaNet (ELN) classification [40]. Adverse events were classified based on the Common Terminology Criteria for Adverse Events (CTCAE) v5.0 [41], except for the occurrence of TLS, for which the Cairo-Bishop classification was employed [30]. Occurrence of AKI was defined by an increase in serum creatinine levels of ≥0.3 mg/dL corresponding to 26 µmol/L or an increase ≥1.5-fold during the first cycle of HMA/VEN [42]. Oliguria was not included in this analysis. The kidney function before VEN was classified based on the eGFR. Cut-offs were applied as described in the CKD nomenclature by KDIGO [43]. KDIGO > 2 (eGFR < 60 mL/min/1.73 m^2^) was defined as CKD. Albuminuria was not taken into consideration due to the lack of data. The following factors were considered as cardiovascular risk factors: arterial hypertension, coronary artery disease, hyperlipidemia, diabetes, smoking, and obesity. 

### 2.5. Statistical Analysis

Median survival times were estimated with the Kaplan–Meier method, median follow-up time with the reverse Kaplan–Meier method. The log-rank test was applied to compare the survival of different subgroups. OS was defined as the time between treatment start and death, EFS between treatment start and refractory disease, relapse or death and RFS from the time of remission to relapse or death. RFS was only measured in patients achieving CR/CRi. If no event had occurred patients were censored as of their last date of follow-up.

The odds ratio (OR) was applied to determine the association of patient characteristics with ORR, AKI or CKD and was calculated based on logistic regression models. The Chi-squared test was used to determine significance of the association. The test was considered significant if *p* < 0.05. Hazard ratios (HR) were calculated to determine the impact of AKI and CKD on survival and were derived from Cox regression models. Univariate and multivariable Cox proportional hazard models were used to evaluate the significance of clinical and molecular characteristics for survival. Parameters with a *p*-value ≤ 0.15 in univariate analysis (UVA) were included in multivariable analysis (MVA). For MVA missing values regarding presence or absence of a complex karyotype and ECOG status were imputed using classification and regression trees (CART) [44]. MVA was performed with the backward elimination procedure. Parameters with a *p*-value ≥ 0.05 were eliminated. The statistical analyses were performed using statistical software environment R, version 4.1.1 using packages mice, survival, cmprsk and forestplot (R Foundation for Statistical Computing, Vienna, Austria), and statistical software package SPSS 29.0 (IBM Corporation, Armonk, NY, USA).

## 3. Results

### 3.1. Patient Characteristics

In total, 253 newly diagnosed patients with AML were enrolled. When excluding intensively treated patients (*n* = 18) and patients without reported renal function (*n* = 105), 130 patients remained for the analysis of the impact of the renal function under VEN treatment (Figure S1). 

Patients received a median of three treatment cycles (range, 1–20). Most commonly, VEN doses of 100 mg (reduction due to co-medication with azoles, *n* = 58; 45%) and 400 mg (*n* = 58; 45%) were used. Detailed treatment characteristics are provided in Table S1. The median age in this cohort was 76 years (range, 27–90 years). ELN risk was favorable, intermediate and adverse in 19 (15%), 24 (19%) and 82 (63%) patients, respectively. A complex karyotype was reported in 26 (20%) patients, and 56 (43%) patients were diagnosed with either secondary or therapy-related AML (Table 1). 

Molecular data was available for 123 patients (95%). In nine patients (7%) no mutation was detected. Genes associated with clonal hematopoiesis were commonly mutated (*ASXL1 n* = 31 (24%), *TET2 n* = 24 (18%), *DNMT3A n* = 20 (15%)). Other frequently detected mutations included *NPM1* (*n* = 26, 20%), *RUNX1* (*n* = 26, 20%) and *TP53* (*n* = 21, 16%). Myelodysplasia-related gene mutations (MRGMs) were detected in 61 patients (47%). Six patients had mutations in driver genes of myeloproliferative neoplasms (MPNs) (*JAK2 n* = 5 (4%), *CALR n* = 1 (1%)) (Table S2). According to the molecular prognostic risk signature (mPRS) classification, 80 (62%), 20 (15%) and 21 (16%) patients were expected to have a higher, intermediate and lower benefit of treatment with HMA/VEN (Table 1). 

The majority of patients (*n* = 96, 74%) had at least one cardiovascular risk factor. Median eGFR before treatment with VEN was 63.1 mL/min/1.73 m^2^ (range, 8.6–126.4 mL/min/1.73 m^2^) and median creatinine was 1.06 mg/dL (range, 0.55–5.68 mg/dL), corresponding to 93.3 µmol/L (range, 48.4–499.8 µmol/L). CKD before VEN was present in 56 (43%) patients (median creatinine 79.2 µmol/L, range 48–499 µmol/L with KDIGO ≤ 2 vs. 125.0 µmol/L, range 87.1–499.8 µmol/L with KDIGO > 2). No other differences in baseline characteristics were observed when comparing patients based on their kidney function before treatment (Table S3). 

### 3.2. Treatment Response and Outcome 

The overall response rate was 45% (*n* = 59) for all patients, with 41 (31%) and 18 (14%) patients achieving CR and CRi. Thirty-nine patients (30%) could not be evaluated for response due to loss to follow-up or death before first response assessment (Table S4). Neither clinical nor molecular characteristics were predictive for ORR. 

**Figure 1:**
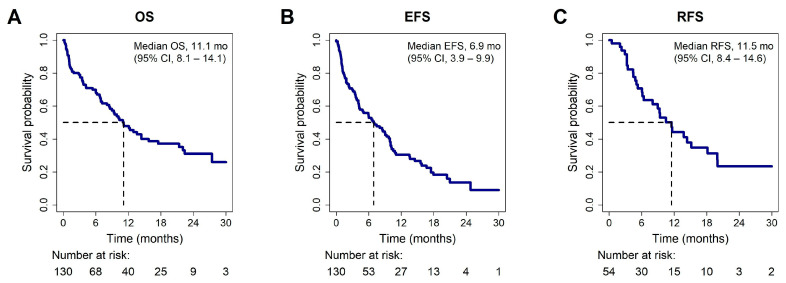
Kaplan–Meier estimates of survival for the entire cohort (*N* = 130). (**A**) Kaplan–Meier estimates for overall survival. (**B**) Kaplan–Meier estimates for event-free survival. (**C**) Kaplan–Meier estimates for relapse-free survival in CR/CRi patients (*n* = 54).

At a median follow-up time of 18.2 months (range, 0.2–35.1 months), the median overall survival (OS) was 11.1 months (95% CI, 8.1–14.1 months) and the median event-free survival (EFS) was 6.9 months (95% CI, 3.9–9.9 months). Patients in CR/CRi had a median relapse-free survival (RFS) of 11.5 months (95% CI, 8.4–14.6 months) (Figure 1). Median duration of response was 7.0 months (range, 0.1–32.7 months).

### 3.3. Association of Pre-Existing Chronic Kidney Disease with Response and Survival

The ORR in the 56 (43%) patients with CKD before VEN was 43%, while 60% of patients with a normal kidney function responded to treatment. In UVA a trend towards a lower ORR was observed, which also persisted in MVA (OR 0.49; 95% CI 0.23–1.05, *p* = 0.07). When combining CR/CRi and MLFS, 49% of patients with CKD and 68% of patients with normal kidney function prior to treatment responded to HMA/VEN (OR 0.45; 95% CI 0.21–0.97, *p* = 0.04), which also remained significant in MVA (OR 0.45; 95% CI 0.21–0.97, *p* = 0.04).

**Figure 2:**
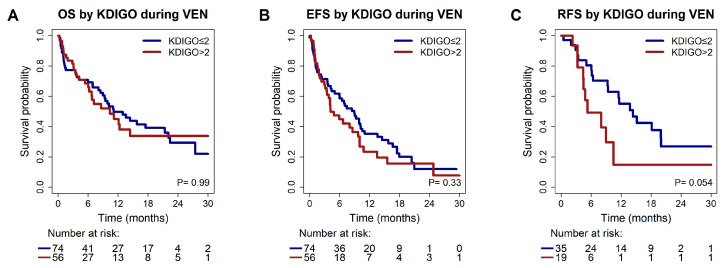
Kaplan–Meier estimates for survival grouped by KDIGO ≤ 2 vs. KDIGO > 2 at the time before treatment start and results of the log-rank test. (**A**) Kaplan–Meier estimates for overall survival. (**B**) Kaplan–Meier estimates for event-free survival. (**C**) Kaplan–Meier estimates for relapse-free survival in CR/CRi patients.

The kidney function before VEN was associated with a median OS of 10.4 vs. 11.1 months in patients with KDIGO > 2 vs. KDIGO ≤ 2 (*p* = 0.99), and a median EFS of 4.2 months vs. 8.9 months in patients with KDIGO > 2 vs. KDIGO ≤ 2 (*p* = 0.3, Figure 2). 

In UVA, a trend towards a shorter RFS was observed with a median RFS of 5.3 vs. 14.4 months in patients with KDIGO > 2 vs. KDIGO ≤ 2 (HR 2.16; 95% CI 1.0–4.8, *p* = 0.06), but CKD was not predictive for RFS in MVA (HR 1.93; 95% CI 0.8–4.8, *p* = 0.15). *TP53* was the only marker that remained predictive for RFS in MVA (HR 2.91; 95% CI 1.2–7.0, *p* = 0.02, Table 2, Figures S2 and S3).

### 3.4. Risk Factors for Acute Kidney Injury During VEN Treatment

In 49 patients (38%) kidney function deteriorated by at least one grade during the first 4 weeks of treatment, with a similar rate between patients with vs. without CKD before treatment start (*n* = 25 [19%] with baseline KDIGO > 2, and *n* = 24 [19%] with baseline KDIGO ≤ 2, HR 1.68; 95% CI 0.82–3.44, *p* = 0.16). No other baseline or treatment characteristics were associated with the occurrence of AKI (Table S5). CKD before VEN was not associated with a higher risk for AKI during treatment, nor were MRGM, MPN-related mutations or CHIP.

Dose ramp-up of VEN was used with similar frequency in patients with vs. without AKI (22 (46%) vs. 47 (61%), *p* = 0.1). LTLS parameters were available in 59 (45%) patients (Table S6). In this subgroup, isolated hyperphosphatemia occurring within the first week after treatment initiation (HR 4.11, 95% CI 1.32–12.80, *p* = 0.01), as well as LTLS according to the Cairo-Bishop classification (HR 8.86, 95% CI 0.99–79.0, *p* = 0.02) were associated with a higher risk of AKI. CTLS parameters were available in 113 (87%) patients. The correlation of a CTLS with AKI did not reach statistical significance (HR 2.46, 95% CI 0.65–9.27, *p* = 0.17). 

### 3.5. Association of Acute Kidney Injury During the First 4 Weeks of VEN Treatment with Response and Survival

The ORR was 43% in patients who developed AKI during treatment, compared to 59% in those with stable kidney function (OR 0.53; 95% CI 0.25–1.15, *p* = 0.11). When MLFS is considered alongside CR/CRi, patients with AKI had a significantly lower response rate in UVA than those without AKI, with response rates of 48% and 67%, respectively (OR 0.45; 95% CI 0.20–0.98, *p* = 0.04). In MVA, a trend toward a lower response rate persisted (OR 0.48; 95% CI 0.22–1.07, *p* = 0.07). The 30-day- and 60-day mortality rates in patients with vs. without AKI were 22% vs. 4% (*p* < 0.001) and 29% vs. 14% (*p* = 0.04), respectively. 

**Figure 3:**
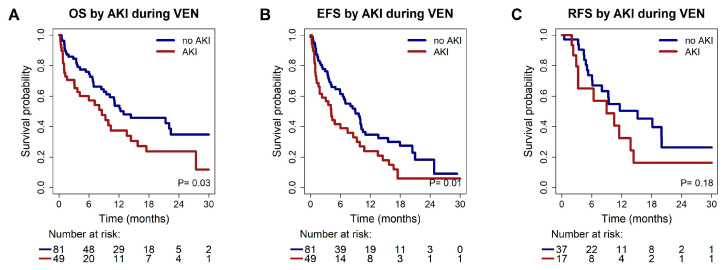
Kaplan-Meier estimates for survival grouped by AKI vs. no AKI during the first treatment cycle of VEN/HMA. (**A**) Kaplan-Meier estimates for overall survival. (**B**) Kaplan-Meier estimates for event-free survival. (**C**) Kaplan Meier estimates for relapse-free survival in CR/CRi patients.

Median OS for patients with AKI was 8.6 months, while it was 12.9 months in patients with a stable kidney function during treatment (HR 1.68, 95% CI 1.04–2.70, *p* = 0.03) (Figure 3).

Median OS did not significantly differ between patients classified with AKI grade I compared to AKI > grade I. In MVA of OS mutated *IDH2* remained as an independent prognostic marker for a superior OS (HR 0.37, 95% CI 0.15–0.92, *p* = 0.03). Mutated *TP53* (HR 2.19, 95% CI 1.25–3.84, *p* = 0.006) and AKI (HR 1.86, 95% CI 1.15–3.02, *p* = 0.01) remained as independent prognostic markers for an inferior OS (Table 2). 

Median EFS in patients with vs. without AKI was 4.2 months (95% CI 2.6–5.7 months) vs. 9.0 months (95% CI 6.3–11.7 months, HR 1.72, 95% CI 1.13–2.63, *p* = 0.01). No significant difference in median EFS between AKI grade I vs. >grade I was observed. In MVA mutated *TP53* (HR 1.91, 95% CI 1.1–3.20, *p* = 0.02) and occurrence of AKI (HR 1.81, 95% CI 1.2–2.8, *p* = 0.007) remained as independent prognostic markers for an inferior EFS (Table 2). 

Median RFS in patients with vs. without AKI was 9.0 months (95% CI 2.1–15.9 months) vs. 15.2 months (95% CI 6.3–24.0 months, *p* = 0.18, Figure 3).

Hazard ratios for OS, EFS and RFS in patients with vs. without AKI were calculated to determine the impact of AKI in different subgroups. AKI was associated with a higher HR for death in patients with de novo AML, favorable ELN2022 risk and wildtype *ASXL1*, *FLT3*-ITD, *IDH1*, *IDH2,* and *RUNX1* (Figure S4). Regarding EFS, AKI was associated with a higher HR in the subgroups mentioned above, as well as patients aged >75 years, male patients, patients with a higher expected benefit according to mPRS, patients without complex karyotype, wildtype *NPM1*, and mutated *TP53* (Figure S5). In the mPRS higher benefit group, AKI was associated with a higher HR for relapse (Figure S6).

### 3.6. Safety

Safety information was available for 113 (87%) patients. The most commonly observed non-hematological adverse events (AE) were of infectious origin. Neutropenic fever was reported in 26 (20%) and pneumonia in 25 (19%) patients. At data cut off 69 patients (53%) had died. The underlying disease was the most common cause of death (*n* = 36, 52%) (Table S7). A significant difference was observed regarding the frequency of sepsis. Thirteen (27%) patients with AKI presented with sepsis, with 10 of these patients dying between days 8 and 242. In contrast, only six patients (7%) with a stable kidney function during VEN treatment presented with sepsis (*p* = 0.01), with two patients dying on days 16 and 204.

## 4. Discussion

With HMA/VEN being broadly applied in AML patients ineligible to receive intensive chemotherapy, it is essential to identify predictors of response and survival and optimize treatment conditions to further improve outcomes. In this analysis, we focused on the assessment of the kidney function before and during treatment with HMA/VEN and its impact on outcome and survival in a cohort of 130 newly diagnosed AML patients. 

Both CKD and AKI were common comorbidities in this cohort. CKD was present in 56 patients (43%). So far, no data on presence of CKD prior to treatment with VEN have been reported. In clinical trials an adequate renal function is commonly listed among the inclusion criteria [45]. Exclusion of patients with impaired kidney function might contribute to the discrepancy in response rates in clinical trials compared to real-world data. In our cohort, CKD neither independently impacted response to HMA/VEN, RFS, EFS nor OS.

In contrast, AKI during HMA/VEN treatment occurred in 49 patients (38%) and was identified as an independent risk factor for OS and EFS. Furthermore, in univariate analysis, a trend towards a lower ORR was observed in the subgroup experiencing AKI. Lower ORR and higher mortality at 30 and 60 days after treatment initiation most likely account for the shorter EFS. 

To our knowledge, the prognostic impact of AKI on outcomes in non-intensive treatment regimens has not been described in previous studies, but our findings are supported by analyses conducted in intensively treated AML patients, showing significantly lower response rates, a higher mortality and a shorter OS in patients with vs. without AKI [46,47]. Short et al. reported two cases of grade 3 AKI (7%) in AML patients receiving frontline therapy with AZA/VEN plus Gilteritinib, but did not describe frequency of lower grade AKI [48]. In our study the predictive value of AKI for OS and EFS was irrespective of the severity of kidney injury, emphasizing the relevance of reporting lower grade AKI. 

Previous reports suggested an increased risk of kidney injury in patients with CHIP or MPN related mutations [25]. We did not observe a significant association between these genetic alterations and the incidence of AKI or CKD in our cohort. However, the number of patients with MPN-associated mutations was low, thus results regarding this subgroup should be interpreted with caution. Additionally, further confounding factors present in our cohort may outweigh the effect of CHIP or MPN on kidney outcomes. Specifically, the nephrotoxic effect of chemotherapy might dominate over the chronic pro-inflammatory process associated with CHIP and MPN mutations.

In contrast to previously described genetic risk factors, which are non-modifiable and only partially addressed by targeted therapies, a decline in kidney function can be identified prior to the onset of overt renal failure through routine monitoring of kidney function. Early detection facilitates the prompt initiation of supportive measures and pharmacologic interventions; however, the impact of such strategies on leukemia outcomes remains to be established in future studies. No baseline characteristics were associated with AKI, and notably neither were treatment characteristics. The frequency of AKI was independent of VEN dose, application of azoles and dose ramp-up. Solely the occurrence of TLS correlated with an increased frequency of AKI. This observation corresponds to an earlier study, in which renal failure was observed significantly more often in patients with LTLS and CTLS than in patients without TLS. In the same cohort, TLS was reported to result in higher rates of death, which in great parts was explained by renal failure [29]. We did not find an association between CKD and TLS prior to treatment and increased risk for AKI. Cancer-related AKI had previously been described as being associated with CKD [49]. However, this analysis was not specific for AML patients undergoing chemotherapy, which might explain why it is not in line with the findings in our cohort. 

Patients with AKI during treatment with HMA/VEN had an increased risk for sepsis and death from sepsis. Together with previous studies, in which AKI was identified as an independent risk factor for severe sepsis and was associated with an increased risk for infection, our study suggests to explore intensified infection management in patients at risk as a potential mitigation strategy. At present, there are no guideline-endorsed immunoprophylactic or immunomodulatory interventions to address AKI-related immune dysfunction [50,51]. Therefore, patients with AKI should be managed with rigorous infection-prevention bundles, including early recognition and prompt treatment of sepsis. Antimicrobial stewardship should be followed, with dosing carefully adjusted to renal function, and nephrotoxic combinations avoided if possible. Supportive strategies such as meticulous catheter and line care, optimal fluid management, and adequate nutritional support are central to reducing infection risk. Although the time intervals between AKI and sepsis were relatively long in some of our patients, a causal association is still possible, as susceptibility for infections remains increased for up to one year after AKI [52,53]. AKI has broad and long-lasting effects on the immune system. It is associated with impaired monocyte cytokine production, elevated plasma cytokine levels, and a pronounced imbalance between pro- and anti-inflammatory transcriptional programs, among other consequences [54,55]. AKI also activates renal sympathetic afferents and central neuroinflammatory pathways, thereby modulating systemic immune responses via neuroimmune circuits such as the cholinergic anti-inflammatory pathway [56]. Sympathetic innervation of the bone marrow is a key regulator of hematopoiesis [57], but to date, no direct evidence demonstrates that AKI alters this neural control, although indirect effects through systemic inflammation and autonomic imbalance are likely. Importantly, the resulting immunocompromised state may persist even after partial or complete remission of AKI, as demonstrated by Grigoryev et al. In addition, the impact of AKI is not confined to the kidney itself but extends to extra-renal organs [54,55]. Pre-existing organ damage caused by AKI may increase the risk of subsequent organ failure and may thereby contribute to the elevated mortality from sepsis.

The increased risk of infections and sepsis might also contribute to the higher early mortality rates seen in patients with AKI. Increased infection susceptibility could be of particular interest, as infectious adverse events are commonly reported in HMA/VEN treated patients, impacting outcome and quality of life [58,59]. 

Limitations of the current study include its design as a registry, which does not control for selection bias. Adverse events are usually underreported in registry trials and this is also the case in the present study. Regarding AKI, the non-availability of urine volume as a diagnostic criterion possibly led to an underestimation of AKI incidence. Due to its retrospective and multicenter set up, availability of further laboratory markers, including albuminuria, and supportive medications apart from the chemotherapeutic agents is limited. In addition, given the small sample size in some molecular subgroups, the statistical power of the analysis regarding these mutations is limited and requires validation in a larger cohort. 

## 5. Conclusions

In summary, CKD before treatment was not predictive for survival; thus, VEN-based treatments remain a feasible option in this subgroup of patients. AKI was a common adverse event and was associated with a higher risk for sepsis and death by sepsis, indicating a potential benefit of intensified infection management. AKI was furthermore identified as an independent risk factor for EFS and OS, suggesting that treatment adjustments and supportive measures should be further evaluated to improve outcomes in VEN-treated AML patients with AKI.

## Figures and Tables

**Table 1:** Treatment and disease characteristics of patients with newly diagnosed AML at time of diagnosis.

Baseline Characteristics	All Patients (*N* = 130)
Age, years Median Range	76 27–90
Sex, *n* (%) Male Female	87 (67) 43 (33)
ECOG performance status, *n* (%) ≤1 >1 Missing data	50 (39) 37 (28) 43 (33)
ICC 2022 classification, *n* (%) AML with recurrent genetic abnormality AML with MRGM AML with MRCA AML with mutated *TP53* AML not otherwise specified Missing data	29 (22) 47 (37) 13 (10) 21 (16) 17 (13) 3 (2)
Type of AML, *n* (%) De novo Secondary or therapy related Missing data	67 (52) 56 (43) 7 (5)
mPRS risk group, *n* (%) Higher benefit Intermediate benefit Lower benefit Missing data	80 (62) 20 (15) 21 (16 9 (7)
ELN 2022 risk group, *n* (%) Favorable/Intermediate Adverse Missing data	43 (33) 82 (63) 5 (4)
Complex karyotype, *n* (%) Yes No Missing data	26 (20) 87 (67) 17 (13)
Prior CMML, *n* (%) Yes No Missing data	9 (7) 105 (81) 16 (12)
Cardiovascular risk factors ^1^, *n* (%) Yes No Missing data	96 (73) 32 (24) 2 (2)
Peripheral blood blasts (%) Median Range Missing data	16 0–99 23
Bone marrow blasts (%) Median Range Missing data	55 10–99 31
WBC count (×10^9^/L) Median Range Missing data	4.6 0.2–171 4
Hemoglobin (g/dL) Median Range Missing data	8.5 4.9–11.7 6
Platelet count (×10^9^/L) Median Range Missing data	42 5–378 6

Abbreviations: ECOG, Eastern Cooperative Oncology Group; ELN, European LeukemiaNet; ICC, International Consensus Classification; mPRS, molecular prognostic risk signature; MRCA myelodysplasia-related cytogenetic abnormalities; MRGM, myelodysplasia-related gene mutations; WBC, white blood cell. ^1^ The following factors were considered as cardiovascular risk factors: arterial hypertension, coronary artery disease, hyperlipidemia, diabetes, smoking, and obesity.

**Table 2:** Multivariable analysis of clinical and molecular markers for OS, RFS, EFS and ORR.

Endpoint	Variables in the Model	Univariate Analysis				Multivariable Analysis		
		HR ^#^	95% CI	*p*		HR ^#^	95% CI	*p*
								
OS	AKI during VEN yes vs. no	1.68	1.0;2.7	0.03		1.86	1.2;3.0	0.01
	Complex karyotype yes vs. no	2.23	1.3;3.8	0.003		1.44	0.7;2.9	0.29
	*IDH2* mut vs. wt	0.39	0.2;1.0	0.05		0.37	0.2;0.9	0.03
	*TP53* mut vs. wt	2.23	1.2;3.8	0.007		2.19	1.3;3.8	0.006
								
EFS	AKI during VEN yes vs. no	1.72	1.1;2.6	0.01		1.81	1.2;2.8	0.007
	Complex karyotype yes vs. no	1.51	1.0;1.5	0.08		1.20	0.7;2.2	0.56
	ECOG > 1 vs. ≤1	1.43	0.9;2.2	0.10		1.49	1.0;2.3	0.06
	*TP53* mut vs. wt	1.78	1.1;3.0	0.03		1.91	1.1;3.2	0.02
								
RFS	ECOG > 1 vs. ≤1	1.77	0.9;3.7	0.13		1.78	0.9;3.7	0.12
	KDIGO > 2 vs. ≤2	2.16	0.97;4.83	0.06		1.93	0.8;4.7	0.15
	*TP53* mut vs. wt	2.91	1.2;7.0	0.02		2.91	1.2;7.0	0.02
								
		OR ^##^	95% CI	*p*		OR ^#^	95% CI	*p*
								
ORR	AKI during VEN yes vs. no	0.53	0.25;1.15	0.11		0.57	0.26;1.26	0.16
	KDIGO > 2 vs. ≤2	0.49	0.23;1.05	0.07		0.49	0.23;1.05	0.07

^#^ Hazard ratios greater than or less than 1 indicate an increased or decreased risk of an event for the first category listed, respectively. ^##^ Odds ratios greater than or less than 1 indicate an increased or decreased probability of an overall response for the first category listed, respectively. Abbreviations: AKI, acute kidney injury; CI, confidence interval; ECOG, Eastern Cooperative Oncology Group; EFS, event-free survival; HR, hazard ratio; KDIGO, Kidney Disease: Improving Global Outcomes; mut; mutated; *p*, *p*-value; OR, odds ratio; ORR, overall response rate; OS, overall survival; RFS, relapse-free survival; VEN, Venetoclax; wt, wildtype.

## Data Availability

Individual patient data will not be made available in order to maintain health information privacy. De-identified information will be shared upon reasonable request to the corresponding author.

## Supplementary Materials

The following supporting information can be downloaded at: https://www.mdpi.com/article/10.3390/cancers17182993/s1, Table S1: Treatment characteristics of patients with newly diagnosed AML at time of diagnosis; Table S2: Molecular characteristics at time of diagnosis; Table S3: Patient and disease characteristics of patients with newly diagnosed AML at time of diagnosis grouped by KDIGO; Table S4: Treatment response after HMA/VEN or LDAC/VEN; Table S5: Patient and disease characteristics of patients with newly diagnosed AML at time of diagnosis grouped by AKI* during the first treatment cycle; Table S6: Kidney function before and during the first treatment cycle; Table S7: Causes of death; Figure S1: Patient selection for analysis; Figure S2: Forest Plot showing results of multivariable analysis of risk factors identified in UVA on OS, EFS and RFS; Figure S3: Kaplan–Meier estimates for survival grouped by risk factors identified in UVA; Figure S4: Prognostic effect of AKI on OS in subgroups; Figure S5: Prognostic effect of AKI on EFS in subgroups; Figure S6: Prognostic effect of AKI on RFS in subgroups.

## Abbreviations

The following abbreviations are used in this manuscript:

AE	Adverse event
AKI	Acute kidney injury
AML	Acute myeloid leukemia
AZA	Azacitidine
CART	Classification and regression trees
CHIP	Clonal hematopoiesis of indeterminate potential
CI	Confidence interval
CKD	Chronic kidney disease
CR	Complete remission
CRi	Complete remission with incomplete blood count recovery
CTCE	Common Terminology Criteria for Adverse Events
CTLS	Clinical tumor lysis syndrome
ECOG	Eastern Cooperative Oncology Group
EFS	Event-free survival
eGFR	Estimated glomerular filtration rate
ELN	European LeukemiaNet
HMA	Hypomethylating agents
HR	Hazard ratio
ICC	International consensus classification
KDIGO	Kidney disease improving global outcomes
KT	karyotype
LDAC	Low-dose cytarabine
LTLS	Laboratory tumor lysis syndrome
MPN	Myeloproliferative neoplasms
mPRS	Molecular prognostic risk signature
MRCA	Myelodysplasia-related cytogenetic abnoramlities
MRGM	Myelodysplasia-related gene mutations
mut	mutated
MVA	Multivariable analysis
OR	Odds ratio
ORR	Overall response rate
OS	Overall survival
*p*	p-value
PK	pharmacokinetics
RFS	Relapse-free survival
TLS	Tumor lysis syndrome
UVA	Univariate analysis
VEN	Venetoclax
VENreg	Venetoclax registry
WBC	White blood cell
wt	Wildtype
