# Supplementary material for: Prognostic Role of Kidney Disease in Newly Diagnosed Acute Myeloid Leukemia Under Venetoclax-Based Low-Intensity Therapy
Source: Cancers (Basel). 2025 Sep 13;17(18):2993. doi: 10.3390/cancers17182993 (PMC12468355; doi:10.3390/cancers17182993)
Supplement: Supplementary file 1 [file cancers-17-02993-s001.zip › cancers-3846577-supplementary.pdf]

**Table S1.** Treatment characteristics of patients with newly diagnosed AML at time of diagnosis.

| Treatment characteristics | All patients (N = 130) |
|---|---|
| VEN dose, n (%) | |
| 100 mg | 58 (45) |
| 400 mg | 58 (45) |
| Other | 9 (6) |
| Missing data | 5 (4) |
| Concomitant azoles, n (%) | |
| Yes | 63 (48) |
| No | 57 (44) |
| Missing data | 10 (8) |
| Dose ramp up, n (%) | |
| Yes | 69 (53) |
| No | 56 (43) |
| Missing data | 5 (4) |
| Duration of VEN per cycle, n (%) | |
| 14d | 43 (33) |
| 28d | 60 (46) |
| Other | 17 (13) |
| Missing data | 10 (8) |
| Number of treatment cycles, n (%) | |
| Median | 3 |
| Range | 1–20 |
| Missing data | 12 |
| Combination partner, n (%) | |
| Azacitidine | 111 (85) |
| Decitabine | 11 (8) |
| LDAC | 6 (5) |
| Missing data | 2 (2) |

Abbreviations: LDAC, low-dose cytarabine; VEN, Venetoclax.

**Table S2.** Molecular characteristics at time of diagnosis.

| Molecular characteristics | All patients (N = 130) |
|---|---|
| *ASXL1*, *n* (%) | |
| Mutated | 31 (24) |
| Wildtype | 90 (69) |
| Missing data | 9 (7) |
| *BCOR*, *n* (%) | |
| Mutated | 12 (9) |
| Wildtype | 92 (71) |
| Missing data | 26 (20) |
| *DNMT3A*, *n* (%) | |
| Mutated | 20 (15) |
| Wildtype | 84 (65) |
| Missing data | 26 (20) |
| *FLT3*-ITD, *n* (%) | |
| Mutated | 10 (8) |
| Wildtype | 111 (85) |
| Missing data | 9 (7) |
| *IDH1*, *n* (%) | |
| Mutated | 11 (8) |
| Wildtype | 110 (85) |
| Missing data | 9 (7) |
| *IDH2*, *n* (%) | |
| Mutated | 18 (14) |

| | |
|---|---|
| Wildtype | 103 (79) |
| Missing data | 9 (7) |
| *NPM1*, *n* (%) | |
| Mutated | 26 (20) |
| Wildtype | 95 (73) |
| Missing data | 9 (7) |
| *NRAS*, *n* (%) | |
| Mutated | 11 (8) |
| Wildtype | 93 (72) |
| Missing data | 26 (20) |
| *KRAS*, *n* (%) | |
| Mutated | 4 (3) |
| Wildtype | 100 (77) |
| Missing data | 26 (20) |
| *RUNX1*, *n* (%) | |
| Mutated | 26 (20) |
| Wildtype | 95 (73) |
| Missing data | 9 (7) |
| *SRSF2*, *n* (%) | |
| Mutated | 18 (14) |
| Wildtype | 86 (66) |
| Missing data | 26 (20) |
| *STAG2*, *n* (%) | |
| Mutated | 11 (8) |
| Wildtype | 93 (72) |
| Missing data | 26 (20) |
| *TET2*, *n* (%) | |
| Mutated | 24 (18) |
| Wildtype | 80 (62) |
| Missing data | 26 (20) |
| *TP53*, *n* (%) | |
| Mutated | 21 (16) |
| Wildtype | 100 (77) |
| Missing data | 9 (7) |

**Table S3.** Patient and disease characteristics of patients with newly diagnosed AML at time of diagnosis grouped by KDIGO.

| **Baseline characteristics** | **KDIGO ≤ 2 (*n* = 74)** | **KDIGO > 2 (*n* = 56)** | ***P*** |
|---|---|---|---|
| Age, years | | | |
| Median | 76 | 76.5 | 0.18 |
| Range | 27–86 | 55–90 | |
| Sex, *n* (%) | | | |
| Male | 46 (62) | 41 (73) | 0.19 |
| Female | 28 (38) | 15 (27) | |
| ECOG performance status, *n* (%) | | | |
| ≤1 | 28 (38) | 22 (39) | 0.94 |
| >1 | 21 (28) | 16 (29) | |
| Missing data | 25 (34) | 18 (32) | |
| ICC 2022 classification, *n* (%) | | | |
| AML with recurrent genetic abnormality | 15 (20) | 14 (25) | |
| AML with MRGM | 27 (35) | 20 (35) | 0.52 |
| AML with MRCA | 10 (14) | 3 (5) | |
| AML with mutated *TP53* | 10 (14) | 11 (20) | |
| AML not otherwise specified | 10 (14) | 7 (13) | |
| Missing data | 2 (3) | 1 (2) | |
| Type of AML, *n* (%) | | | 0.86 |
| De novo | 37 (50) | 30 (54) | |

| | | | |
|---|---|---|---|
| Secondary or therapy related | 30 (41) | 26 (46) | |
| Missing data | 7 (9) | - | |
| mPRS risk group, *n*(%) | | | |
| Higher benefit | 50 (67) | 30 (54) | 0.13 |
| Intermediate benefit | 8 (11) | 12 (21) | |
| Lower benefit | 10 (14) | 11 (20) | |
| Missing data | 6 (8) | 3 (5) | |
| ELN 2022 risk group, *n* (%) | | | |
| Favorable/Intermediate | 24 (32) | 19 (34) | 0.98 |
| Adverse | 46 (63) | 36 (64) | |
| Missing data | 4 (5) | 1 (2) | |
| Complex karyotype, *n* (%) | | | |
| Yes | 13 (18) | 13 (23) | 0.64 |
| No | 48 (64) | 39 (70) | |
| Missing data | 13 (18) | 4 (7) | |
| Peripheral blood blasts (%) | | | |
| Median | 21.8 | 10.0 | 0.40 |
| Range | 0–99 | 0–98 | |
| Missing data | 14 | 9 | |
| Bone marrow blasts (%) | | | |
| Median | 54 | 60.0 | 0.93 |
| Range | 0.23–98 | 0.22–99 | |
| Missing data | 17 | 14 | |
| WBC count (x10⁹/L) | | | |
| Median | 4.2 | 5.3 | 0.38 |
| Range | 0.2–145 | 0.6–171 | |
| Missing data | 3 | 1 | |
| Hemoglobin (g/dL) | | | |
| Median | 8.6 | 8.5 | 0.92 |
| Range | 4.9–11.7 | 6.4–11.4 | |
| Missing data | 4 | 2 | |
| Platelet count (x10⁹/L) | | | |
| Median | 47.0 | 38.0 | 0.92 |
| Range | 5–378 | 5–314 | |
| Missing data | 3 | 3 | |
| Creatinine (mg/dL) | | | |
| Median | 0.9 | 1.42 | <0.001 |
| Range | 0.55–1.23 | 0.99–5.68 | |
| Missing data | 2 | - | |
| Cardiovascular risk factors, *n* (%) | | | |
| Yes | 50 (67) | 46 (82) | 0.1 |
| No | 22 (30) | 10 (18) | |
| Missing data | 2 (3) | - | |
| AKI* during C1, *n* (%) | | | |
| Yes | 24 (32) | 25 (45) | 0.16 |
| No | 50 (68) | 31 (55) | |
| Clinical TLS, *n* (%) | | | |
| Yes | 4 (5) | 6 (11) | 0.29 |
| No | 59 (80) | 44 (78) | |
| Missing data | 11 (15) | 6 (11) | |
| Laboratory TLS, *n* (%) | *n* = 22 | *n* = 37 | |
| Yes | 2 (9) | 5 (14) | 0.61 |
| No | 20 (91) | 32 (86) | |

Abbreviations: AKI, acute kidney injury; C1, cycle 1; ECOG, Eastern Cooperative Oncology Group; ELN, European LeukemiaNet; ICC, International Consensus Classification; KDIGO, Kidney Disease: Improving Global Outcomes; mPRS, molecular prognostic risk signature; MRCA myelodysplasia-related cytogenetic abnormalities; MRGM, myelodysplasia-related gene mutations; TLS, tumor lysis syndrome; WBC, white blood cell. *AKI: acute kidney injury; defined by an increase in

serum creatinine of ≥0.3 mg/dL or ≥1.5× compared to the baseline concentration during the first treatment cycle of HMA/VEN.

**Table S4.** Treatment response after HMA/VEN or LDAC/VEN.

| Survival characteristics | All patients $N = 130$ (%) |
|---|---|
| Best response, *n* (%) | |
| CR | 41 (31) |
| CRi | 18 (14) |
| MLFS | 8 (6) |
| SD/RD/PD | 24 (19) |
| Died before first assessment | 23 (18) |
| Missing data | 16 (12) |
| ORR, *n* (%) | 59 (45) |

Abbreviations: CR, complete remission; CRi, complete remission with incomplete blood count recovery. MLFS, morphologic leukemia free state; ORR, overall response rate; PD, progressive disease; RD, refractory disease; SD, stable disease.

**Table S5.** Patient and disease characteristics of patients with newly diagnosed AML at time of diagnosis grouped by AKI* during the first treatment cycle.

| Baseline characteristics | No AKI ($n = 81$) | AKI ($n = 49$) | *P* |
|---|---|---|---|
| Age, years | | | |
| Median | 75 | 76 | 0.99 |
| Range | 27–90 | 55–86 | |
| Sex, *n* (%) | | | |
| Male | 50 (62) | 37 (76) | 0.11 |
| Female | 31 (38) | 12 (24) | |
| ECOG performance status, *n* (%) | | | |
| ≤1 | 30 (37) | 20 (40) | 0.84 |
| >1 | 23 (28) | 14 (29) | |
| Missing data | 28 (35) | 15 (31) | |
| ICC 2022 classification, *n* (%) | | | |
| AML with recurrent genetic abnormality | 17 (21) | 12 (24) | 0.9 |
| AML with MRGM | 30 (37) | 17 (36) | |
| AML with MRCA | 9 (11) | 4 (8) | |
| AML with mutated TP53 | 13 (16) | 8 (16) | |
| AML not otherwise specified | 9 (11) | 8 (16) | |
| Missing data | 3 (4) | - | |
| Type of AML, *n* (%) | | | |
| De novo | 43 (53) | 24 (49) | 0.43 |
| Secondary or therapy related | 32 (40) | 24 (49) | |
| Missing data | 6 (7) | 1 (2) | |
| mPRS risk group, *n*(%) | | | |
| Higher benefit | 53 (66) | 27 (56) | 0.40 |
| Intermediate benefit | 10 (12) | 10 (20) | |
| Lower benefit | 13 (16) | 8 (16) | |
| Missing data | 5 (6) | 4 (8) | |
| ELN 2022 risk group, *n* (%) | | | |
| Favorable/Intermediate | 27 (33) | 16 (33) | 0.74 |
| Adverse | 49 (61) | 33 (67) | |
| Missing data | 5 (6) | - | |
| Complex karyotype, *n* (%) | | | |
| Yes | 14 (17) | 12 (24) | 0.45 |
| No | 54 (67) | 33 (68) | |
| Missing data | 13 (16) | 4 (8) | |
| Peripheral blood blasts | | | 0.73 |

| | | | |
|---|---|---|---|
| Median (%) | 20.0 | 12.6 | |
| Range (%) | 0–99 | 0–97.3 | |
| Missing data, *n* (%) | 14 (11) | 9 (7) | |
| Bone marrow blasts | | | |
| Median (%) | 52.0 | 60.0 | 0.79 |
| Range (%) | 0.22–98 | 0.23–99 | |
| Missing data, *n* (%) | 19 (15) | 12 (9) | |
| WBC count (x10$^9$/L) | | | |
| Median | 4.9 | 4.3 | 0.56 |
| Range | 0.24–171 | 0.2–121 | |
| Missing data, *n* (%) | 2 (2) | 2 (2) | |
| Hemoglobin (g/L) | | | |
| Median | 8.5 | 8.5 | 0.40 |
| Range | 4.9–11.7 | 6.6–11.7 | |
| Missing data, *n* (%) | 3 (2) | 3 (2) | |
| PLT count (x10$^9$/L) | | | |
| Median | 42.5 | 39.0 | 0.94 |
| Range | 5–290 | 7–378 | |
| Missing data, *n* (%) | 3 (2) | 3 (2) | |
| Creatinine (mg/dL) | | | |
| Median | 0.99 | 1.2 | 0.02 |
| Range | 0.55–4.28 | 0.55–5.68 | |
| Missing data, *n* (%) | 1 (1) | 1 (1) | |
| Cardiovascular risk factors, *n* (%) | | | |
| Yes | 59 (73) | 37 (76) | 0.67 |
| No | 21 (26) | 11 (22) | |
| Missing data | 1 (1) | 1 (2) | |
| KDIGO before VEN, *n* (%) | | | |
| ≤2 | 50 (62) | 24 (49) | 0.16 |
| >2 | 31 (38) | 25 (51) | |
| Clinical TLS, *n* (%) | | | |
| Yes | 4 (5) | 6 (12) | 0.17 |
| No | 64 (79) | 39 (80) | |
| Missing data | 13 (16) | 4 (8) | |
| Laboratory TLS, *n* (%) | *n* = 32 | *n* = 27 | |
| Yes | 1 (3) | 6 (22) | 0.02 |
| No | 31 (97) | 21 (78) | |

Abbreviations: AKI, acute kidney injury; C1, cycle 1; ECOG, Eastern Cooperative Oncology Group; ELN, European LeukemiaNet; ICC, International Consensus Classification; KDIGO, Kidney Disease: Improving Global Outcomes; mPRS, molecular prognostic risk signature; MRCA myelodysplasia-related cytogenetic abnormalities; MRGM, myelodysplasia-related gene mutations; PLT, platelet; TLS, tumor lysis syndrome; WBC, white blood cell. *AKI: acute kidney injury; defined by an increase in serum creatinine of ≥0.3 mg/dL or ≥1.5× compared to the baseline concentration.

**Table S6.** Kidney function before and during the first treatment cycle.

| Kidney function | All patients (*N* = 130) |
|---|---|
| Creatinine before VEN (mg/dL) | |
| Median | 1.06 |
| Range | 0.55–5.68 |
| Missing data | 2 (2) |
| eGFR before VEN (mL/min/1.73m$^2$) | |
| Median | 63.1 |
| Range | 8.6–126.4 |
| Missing data | 2 (2) |
| KDIGO before VEN, *n* (%) | |
| 1 | 19 (15) |
| 2 | 55 (43) |

| | |
|---|---|
| 3a | 29 (22) |
| 3b | 16 (12) |
| 4 | 8 (6) |
| 5 | 3 (2) |
| Clinical TLS, *n* (%) | |
| Yes | 10 (8) |
| No | 103 (79) |
| Missing data | 17 (13) |
| Laboratory TLS, *n* (%) | *N* = 59 |
| Yes | 7 (12) |
| No | 52 (88) |
| Hyperkalemia during C1, *n* (%) | *N* = 59 |
| Yes | 6 (10) |
| No | 53 (90) |
| Hyperphosphatemia during C1, *n* (%) | *N* = 57 |
| Yes | 22 (39) |
| No | 35 (61) |
| Hypocalcemia during C1, *n* (%) | *N* = 59 |
| Yes | 4 (7) |
| No | 55 (93) |
| Hyperuricemia during C1, *n* (%) | *N* = 49 |
| Yes | 4 (8) |
| No | 45 (92) |

Abbreviations: C1, cycle 1; eGFR, estimated glomerular filtration rate; KDIGO, Kidney Disease: Improving Global Outcomes; TLS, tumor lysis syndrome.

**Table S7.** Causes of death.

| **Cause of death** | ***N* = 69** |
|---|---|
| Underlying disease, *n* (%) | 37 (53) |
| Infection | 15 (22) |
| Other | 2 (3) |
| Missing data | 15 (22) |

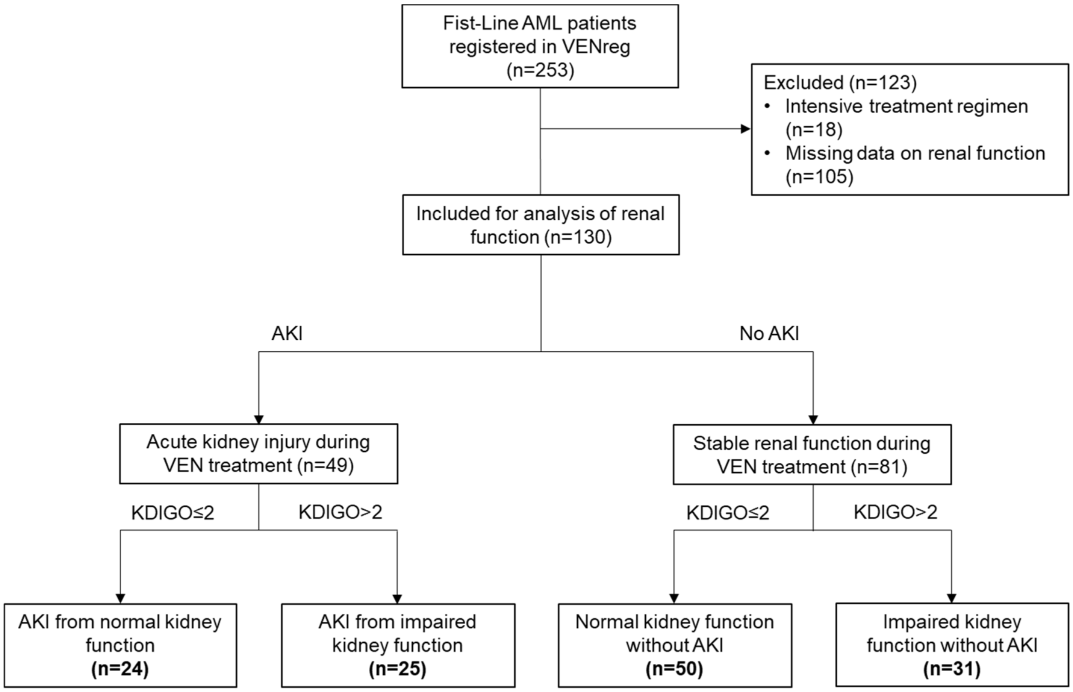


**Figure S1.** Patient selection for analysis.

Abbreviations: AKI, acute kidney injury; AML, acute myeloid leukemia; KDIGO, Kidney Disease: Improving Global Outcomes; VEN, Venetoclax.

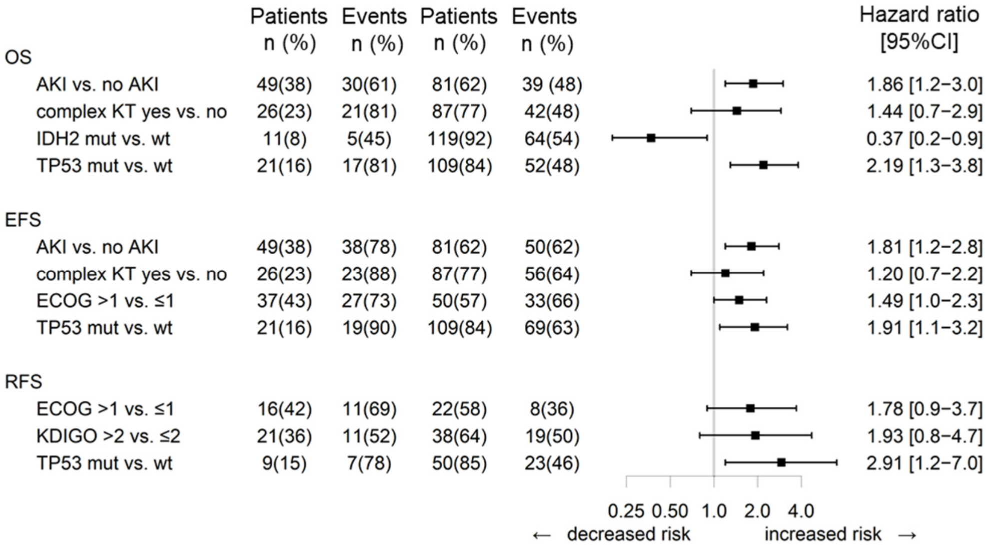


**Figure S2.** Forest plot showing results of multivariable analysis of risk factors identified in UVA on OS, EFS and RFS.

Abbreviations: AKI, acute kidney injury; ECOG, Eastern Cooperative Oncology Group; EFS, event-free survival; KDIGO, Kidney Disease: Improving Global Outcomes; KT, karyotype; OS, overall survival; RFS, relapse-free survival.

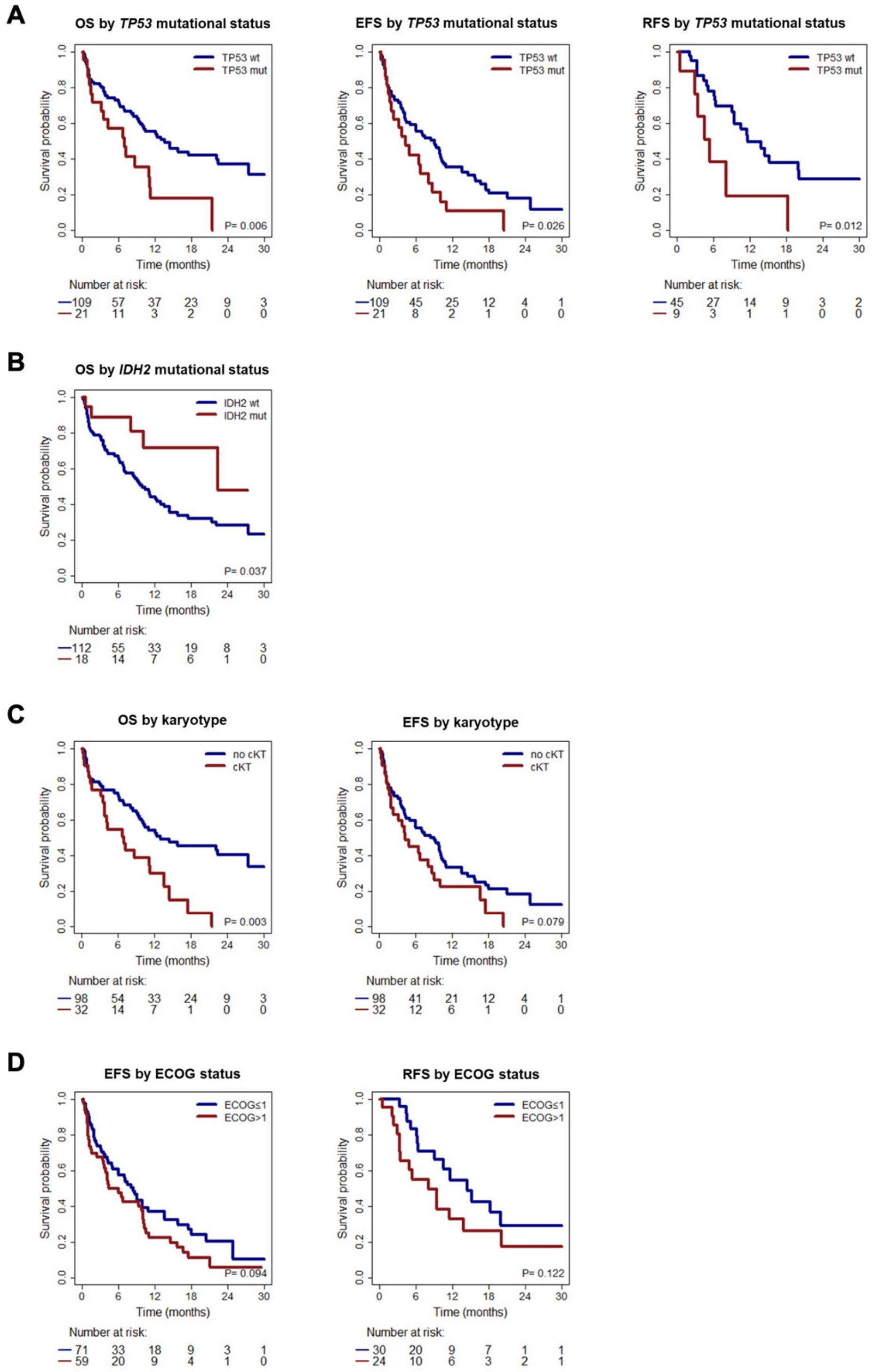


**Figure S3.** Kaplan-Meier estimates for survival grouped by risk factors identified in UVA. (A) Kaplan-Meier estimates for OS, EFS and RFS grouped by mutational status of *TP53.* (B) Kaplan-Meier estimates for OS grouped by mutational status of *IDH2.* (C) Kaplan-Meier estimates for OS and EFS grouped by complexity of karyotype. (D) Kaplan-Meier estimates for EFS and RFS grouped by ECOG status.

Abbreviations: cKT, complex karyotype; ECOG, Eastern Cooperative Oncology Group; EFS, event-free survival; KDIGO, Kidney Disease: Improving Global Outcomes; mut, mutated; OS, overall survival; RFS, relapse-free survival; wt, wildtype.

| Characteristic | Patients n (%) | Events n (%) | Patients n (%) | Events n (%) | Hazard ratio [95%CI] |
|---|---|---|---|---|---|
| Age | | | | | |
| age≤75 | 22(35) | 16(73) | 41(65) | 21(51) | 1.51 [0.79−2.91] |
| age>75 | 27(40) | 14(52) | 40(60) | 18(45) | 1.81 [0.89−2.67] |
| ECOG | | | | | |
| ≤1 | 20(40) | 13(65) | 30(60) | 15(50) | 1.99 [0.94−2.2] |
| >1 | 14(38) | 8(57) | 23(62) | 14(61) | 1.16 [0.49−1.78] |
| AML type | | | | | |
| de novo | 24(36) | 14(58) | 43(64) | 17(40) | 2.78 [1.34−2.76] |
| sAML/tAML | 24(43) | 15(63) | 32(57) | 21(66) | 0.88 [0.45−2.72] |
| Gender | | | | | |
| male | 37(43) | 22(59) | 50(57) | 23(46) | 1.73 [0.96−3.12] |
| female | 12(28) | 8(67) | 31(72) | 16(52) | 1.48 [0.63−1.49] |
| mPRS | | | | | |
| higher benefit | 27(34) | 17(63) | 53(66) | 23(43) | 2.22 [0.86−5.74] |
| intermediate benefit | 10(50) | 3(30) | 10(50) | 4(40) | 0.64 [0.10−4.10] |
| lower benefit | 8(38) | 7(88) | 13(62) | 10(77) | 2.10 [0.18−24.60] |
| ELN2022 | | | | | |
| favorable | 16(37) | 11(69) | 27(63) | 8(30) | 3.45 [1.38−1.62] |
| intermediate/adverse | 33(40) | 19(58) | 49(60) | 29(59) | 1.24 [0.7−3.22] |
| Complex karyotype | | | | | |
| no | 33(38) | 18(55) | 54(62) | 24(44) | 1.75 [0.95−3.26] |
| yes | 12(46) | 10(83) | 14(84) | 11(79) | 1.55 [0.64−1.75] |
| Cardiovascular risk factors | | | | | |
| no | 11(34) | 7(64) | 21(66) | 13(62) | 2.27 [0.87−1.9] |
| yes | 37(39) | 22(59) | 59(61) | 25(42) | 1.67 [0.94−3.97] |
| AlloHSCT after VEN | | | | | |
| no | 44(38) | 26(59) | 73(62) | 33(45) | 1.62 [0.97−4.71] |
| yes | 2(29) | 2(100) | 5(71) | 4(80) | 7.48 [0.66−2.2] |
| ASXL1 | | | | | |
| wildtype | 37(37) | 24(65) | 62(63) | 27(44) | 2.08 [1.2−3.62] |
| mutated | 12(39) | 6(50) | 19(61) | 12(63) | 0.96 [0.35−1.6] |
| FLT3-ITD | | | | | |
| wildtype | 43(36) | 27(63) | 77(64) | 38(49) | 1.65 [1.01−4.71] |
| mutated | 6(60) | 3(50) | 4(40) | 1(25) | 4.01 [0.38−6.91] |
| IDH1 | | | | | |
| wildtype | 45(38) | 29(64) | 74(62) | 35(47) | 1.93 [1.18−4.16] |
| mutated | 4(36) | 1(25) | 7(64) | 4(57) | 0.35 [0.04−4.1] |
| IDH2 | | | | | |
| wildtype | 43(38) | 29(67) | 69(62) | 35(51) | 1.99 [1.21−4.26] |
| mutated | 6(33) | 1(17) | 12(67) | 4(33) | 0.31 [0.03−6.9] |
| NPM1 | | | | | |
| wildtype | 38(37) | 24(63) | 66(63) | 34(52) | 1.48 [0.88−3.5] |
| mutated | 11(42) | 6(55) | 15(58) | 5(33) | 3.36 [0.98−1.52] |
| RUNX1 | | | | | |
| wildtype | 37(36) | 24(65) | 67(64) | 33(49) | 1.96 [1.15−3.33] |
| mutated | 12(46) | 6(50) | 14(54) | 6(43) | 0.98 [0.3−1.266] |
| TP53 | | | | | |
| wildtype | 41(38) | 23(56) | 68(62) | 29(43) | 1.68 [0.97−4.91] |
| mutated | 8(38) | 7(88) | 13(62) | 10(77) | 2.03 [0.75−8.5] |

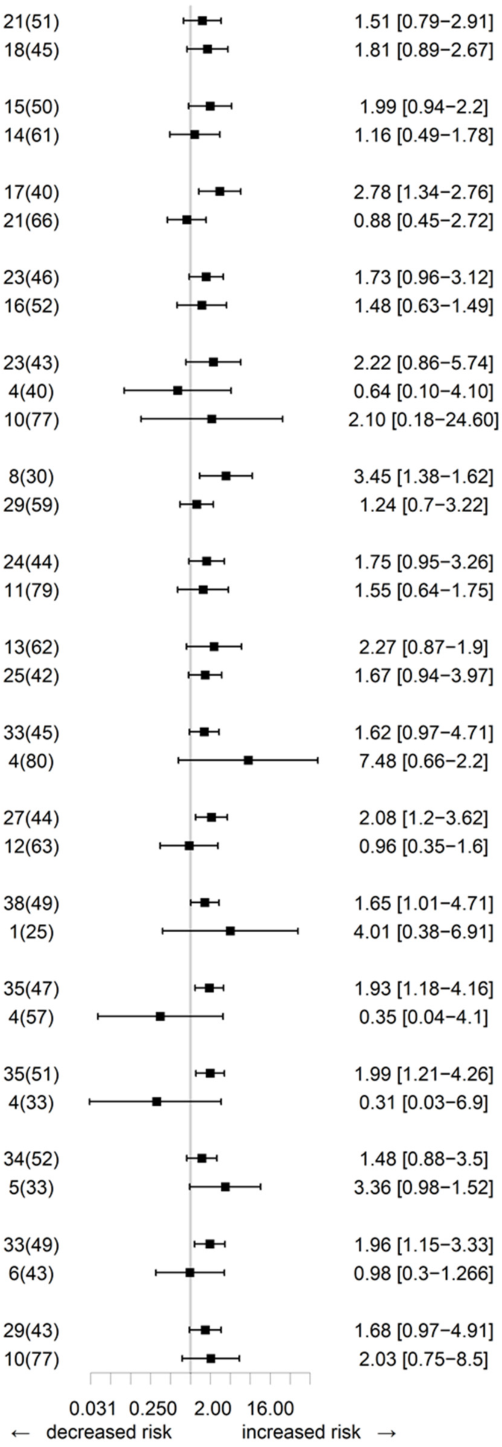


**Figure S4.** Prognostic effect of AKI on OS in subgroups.

Abbreviations: AKI, acute kidney injury; AlloHSCT, allogeneic hematopoietic stem cell transplantation; ECOG, Eastern Cooperative Oncology Group; ELN, European LeukemiaNet; mPRS; molecular prognostic risk signature; OS, overall survival; sAML, secondary AML; tAML, therapy related AML.

| Characteristic | Patients n (%) | Events n (%) | Patients n (%) | Events n (%) | Hazard ratio [95%CI] |
|---|---|---|---|---|---|
| Age | | | | | |
| age≤75 | 22(35) | 19(86) | 41(65) | 27(66) | 1.54 [0.84−2.81] |
| age>75 | 27(40) | 19(70) | 40(60) | 23(58) | 2.16 [1.16−4.00] |
| ECOG | | | | | |
| ≤1 | 20(40) | 15(75) | 30(60) | 18(60) | 2.00 [1.00−4.03] |
| >1 | 14(38) | 11(79) | 23(62) | 16(70) | 1.77 [0.81−3.88] |
| AML type | | | | | |
| de novo | 24(36) | 18(75) | 43(64) | 25(58) | 2.38 [1.27−4.46] |
| sAML/tAML | 24(43) | 19(79) | 32(57) | 24(75) | 1.09 [0.60−1.99] |
| Gender | | | | | |
| male | 37(43) | 28(76) | 50(57) | 30(60) | 1.96 [1.16−3.31] |
| female | 12(28) | 10(83) | 31(72) | 20(65) | 1.31 [0.61−2.80] |
| mPRS | | | | | |
| higher benefit | 27(34) | 21(78) | 53(66) | 28(53) | 3.13 [1.09−8.98] |
| intermediate benefit | 10(50) | 5(50) | 10(50) | 7(70) | 0.43 [0.07−2.68] |
| lower benefit | 8(38) | 8(100) | 13(62) | 11(85) | NA |
| ELN2022 | | | | | |
| favorable | 16(37) | 12(75) | 27(63) | 11(41) | 2.48 [1.07−5.77] |
| intermediate/adverse | 33(40) | 26(79) | 49(60) | 35(71) | 1.57 [0.94−2.62] |
| Complex karyotype | | | | | |
| no | 33(38) | 24(73) | 54(62) | 32(59) | 1.94 [1.13−3.33] |
| yes | 12(46) | 11(92) | 14(54) | 12(86) | 1.51 [0.65−3.51] |
| Cardiovascular risk factors | | | | | |
| no | 11(34) | 11(100) | 21(66) | 14(67) | 2.21 [0.94−5.20] |
| yes | 37(39) | 28(76) | 59(61) | 35(59) | 1.59 [0.97−2.62] |
| AlloHSCT after VEN | | | | | |
| no | 44(38) | 43(98) | 73(62) | 34(47) | 1.66 [1.06−2.61] |
| yes | 2(29) | 2(100) | 5(71) | 4(80) | 3.50 [0.49−25.27] |
| ASXL1 | | | | | |
| wildtype | 37(37) | 30(81) | 62(63) | 35(56) | 1.89 [1.16−3.08] |
| mutated | 12(39) | 8(67) | 19(61) | 15(79) | 1.28 [0.54−3.04] |
| FLT3-ITD | | | | | |
| wildtype | 43(36) | 34(79) | 77(64) | 48(62) | 1.78 [1.15−2.78] |
| mutated | 6(60) | 4(67) | 4(40) | 2(50) | 3.87 [0.43−35.10] |
| IDH1 | | | | | |
| wildtype | 45(38) | 35(78) | 74(62) | 45(61) | 1.81 [1.16−2.82] |
| mutated | 4(36) | 3(75) | 7(64) | 5(71) | 1.11 [0.26−4.79] |
| IDH2 | | | | | |
| wildtype | 43(38) | 33(77) | 69(62) | 44(64) | 1.71 [1.09−2.69] |
| mutated | 6(33) | 5(83) | 12(67) | 6(50) | 1.93 [0.55−6.73] |
| NPM1 | | | | | |
| wildtype | 38(37) | 32(84) | 66(63) | 44(67) | 1.71 [1.08−2.70] |
| mutated | 11(42) | 6(55) | 15(58) | 6(40) | 2.60 [0.78−8.72] |
| RUNX1 | | | | | |
| wildtype | 37(36) | 29(78) | 67(64) | 42(63) | 1.81 [1.12−2.92] |
| mutated | 12(46) | 9(75) | 14(54) | 8(57) | 1.60 [0.60−4.24] |
| TP53 | | | | | |
| wildtype | 41(38) | 30(73) | 68(62) | 39(57) | 1.62 [1.00−2.61] |
| mutated | 8(38) | 8(100) | 13(62) | 11(85) | 3.02 [1.11−8.19] |

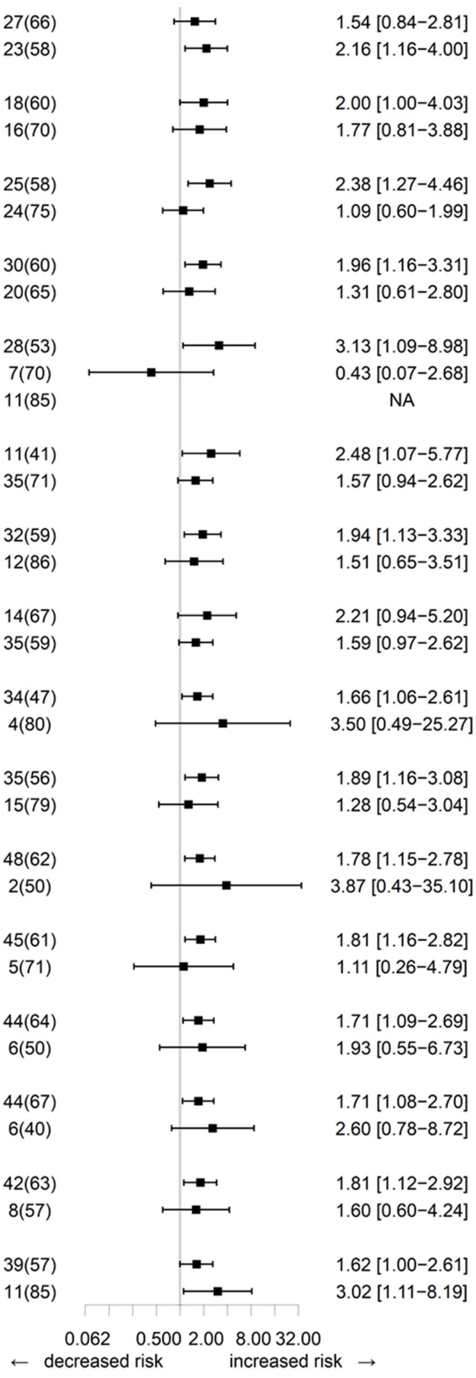


**Figure S5.** Prognostic effect of AKI on EFS in subgroups.

Abbreviations: AKI, acute kidney injury; AlloHSCT, allogeneic hematopoietic stem cell transplantation; ECOG, Eastern Cooperative Oncology Group; EFS; event-free survival; ELN, European LeukemiaNet; mPRS, molecular prognostic risk signature; sAML, secondary AML; tAML, therapy related AML.

| Characteristic | Patients n (%) | Events n (%) | Patients n (%) | Events n (%) | Hazard ratio [95%CI] |
|---|---|---|---|---|---|
| Age | | | | | |
| age≤75 | 6(26) | 5(83) | 17(74) | 9(53) | 2.03 [0.61−6.71] |
| age>75 | 11(35) | 6(55) | 20(65) | 10(50) | 1.57 [0.57−4.36] |
| ECOG | | | | | |
| ≤1 | 5(26) | 2(74) | 14(74) | 6(43) | 1.13 [0.23−5.65] |
| >1 | 5(31) | 4(69) | 11(69) | 8(73) | 1.75 [0.50−6.16] |
| AML type | | | | | |
| de novo | 8(27) | 5(63) | 22(73) | 12(55) | 1.32 [0.45−3.89] |
| sAML/tAML | 8(42) | 5(63) | 11(58) | 6(55) | 1.26 [0.38−4.23] |
| Gender | | | | | |
| male | 11(34) | 7(64) | 21(66) | 11(52) | 2.42 [0.91−6.42] |
| female | 6(27) | 4(67) | 16(73) | 8(50) | 0.96 [0.29−3.22] |
| mPRS | | | | | |
| higher benefit | 9(27) | 6(67) | 24(73) | 6(25) | 6.00 [1.13−31.74] |
| intermediate benefit | 7(54) | 3(43) | 6(46) | 5(83) | 0.15 [0.01−2.06] |
| lower benefit | 1(11) | 1(100) | 8(89) | 6(75) | NA |
| ELN2022 | | | | | |
| favorable | 5(33) | 3(60) | 10(67) | 2(20) | 2.10 [0.33−13.40] |
| intermediate/adverse | 12(32) | 8(67) | 26(68) | 16(62) | 1.25 [0.53−2.93] |
| Complex karyotype | | | | | |
| no | 13(35) | 9(69) | 24(65) | 12(50) | 2.01 [0.84−4.81] |
| yes | 2(20) | 1(50) | 8(80) | 6(75) | 0.65 [0.07−6.11] |
| Cardiovascular risk factors | | | | | |
| no | 10(71) | 7(70) | 4(29) | 3(75) | 1.41 [0.35−5.72] |
| yes | 12(31) | 7(58) | 27(69) | 12(44) | 1.30 [0.50−3.35] |
| AlloHSCT after VEN | | | | | |
| no | 16(33) | 10(63) | 32(67) | 14(44) | 1.61 [0.71−3.63] |
| yes | 1(25) | 1(100) | 3(75) | 3(100) | 2.45 [0.15−39.72] |
| ASXL1 | | | | | |
| wildtype | 13(34) | 8(62) | 25(66) | 11(44) | 1.52 [0.61−3.80] |
| mutated | 4(25) | 3(75) | 12(75) | 8(67) | 1.53 [0.40−5.81] |
| FLT3-ITD | | | | | |
| wildtype | 13(27) | 9(69) | 35(73) | 18(51) | 1.60 [0.71−3.57] |
| mutated | 4(67) | 2(50) | 2(33) | 1(50) | 1.41 [0.13−15.84] |
| IDH1 | | | | | |
| wildtype | 15(31) | 9(60) | 34(69) | 17(50) | 1.30 [0.58−2.92] |
| mutated | 2(40) | 2(100) | 3(60) | 2(67) | NA |
| IDH2 | | | | | |
| wildtype | 12(27) | 7(58) | 32(73) | 18(56) | 1.01 [0.42−2.43] |
| mutated | 5(50) | 4(80) | 5(50) | 1(20) | NA |
| NPM1 | | | | | |
| wildtype | 13(30) | 10(77) | 31(70) | 18(58) | 1.65 [0.76−3.59] |
| mutated | 4(40) | 1(25) | 6(60) | 1(17) | 1.41 [0.09−22.64] |
| RUNX1 | | | | | |
| wildtype | 13(31) | 8(62) | 29(69) | 15(52) | 1.37 [0.58−3.24] |
| mutated | 4(33) | 3(75) | 8(67) | 4(50) | 1.81 [0.40−8.17] |
| TP53 | | | | | |
| wildtype | 16(36) | 10(63) | 29(64) | 13(45) | 1.61 [0.71−3.70] |
| mutated | 1(11) | 1(100) | 8(89) | 6(75) | 7.48 [0.47−119.82] |

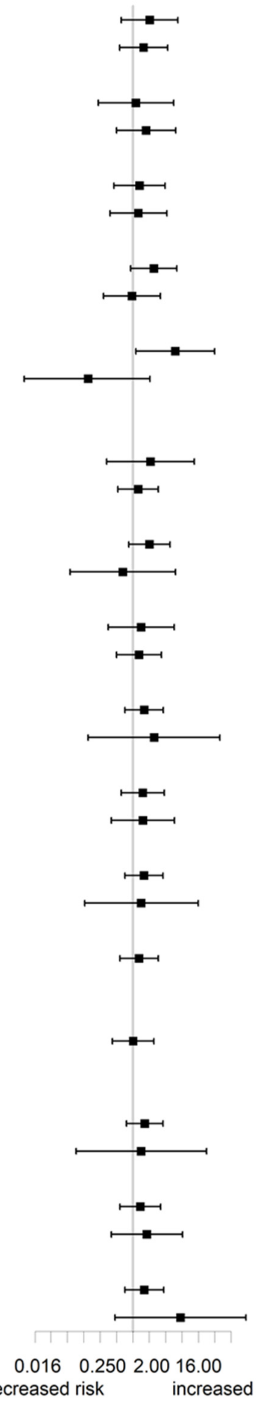


**Figure S6.** Prognostic effect of AKI on RFS in subgroups.
Abbreviations: AKI, acute kidney injury; AlloHSCT, allogeneic hematopoietic stem cell transplantation; ECOG, Eastern Cooperative Oncology Group; ELN, European LeukemiaNet; mPRS, molecular prognostic risk signature; RFS, relapse-free survival; sAML, secondary AML; tAML, therapy related AML.
